# Subcutaneous emphysema and pneumomediastinum in child with asthma revealing occult foreign body aspiration: a case report

**DOI:** 10.1186/s13256-019-2076-x

**Published:** 2019-05-26

**Authors:** Mounir Bourrous, Widad Lahmini, Hassan Nouri, Nouzha Haimeur

**Affiliations:** 1 Department of Paediatric Emergency, UHC Mohamed VI, Cadi Ayyad University, Faculty of medicine and pharmacy, PO Box: 7010, Sidi Abbad Street, 40000 Marrakech, Morocco; 2 Department of ORL, UHC Mohamed VI, Cadi Ayyad University, Marrakech, Morocco; 3 Department of Anesthesia and Critical Care, UHC Mohamed VI, Cadi Ayyad University, Marrakech, Morocco

**Keywords:** Foreign body aspiration, Child, Pneumomediastinum, Subcutaneous emphysema

## Abstract

**Background:**

Exacerbations of asthma constitute the most common cause of pneumomediastinum and subcutaneous emphysema in children. Foreign body aspiration is a rare cause of pneumomediastinum and subcutaneous emphysema. Foreign body aspiration leading to the occurrence of pneumomediastinum in a child with asthma may go unnoticed and be wrongly attributed to asthma, which leads to delayed diagnosis as well as to life-threatening and long-term complications.

**Case presentation:**

We describe a case of a 6-year-old Moroccan boy with asthma who was admitted to our emergency department for acute dyspnea and persistent dry cough. The patient was initially treated as having acute asthma exacerbation. Owing to insufficient clinical and radiographic improvement with asthma treatment, a rigid bronchoscopy under general anesthesia was performed. A pumpkin seed was removed from the left main bronchus. Clinical and radiographic improvement was achieved after foreign body extraction.

**Conclusions:**

This case emphasizes that the possibility of foreign body aspiration should always and carefully be considered by the emergency physician when faced with a child with asthma presenting with pneumomediastinum and subcutaneous emphysema as an important differential diagnosis even in the absence of a history of foreign body aspiration.

## Introduction

Pneumomediastinum (PM) is defined as the presence of free air in the mediastinum. Caused by a perforation of the tracheobronchial tree, it occurs in any condition that creates a gradient between intra-alveolar and perivascular interstitial pressures. Subcutaneous emphysema (SCE) results when air dissects along planes of the mediastinum to the subcutaneous tissues of the thorax, neck, and upper limbs. PM rarely occurs in children and is generally a benign entity that requires supportive care [1, 2]. The most common cause of PM in children is exacerbations of asthma [1–3]. PM and SCE are rare presentations of foreign body (FB) aspiration. In this report, we describe a case of a child with asthma who presented with PM and SCE, which were treated initially as an exacerbation of asthma and later found to be induced by FB aspiration. This case emphasizes the difficulties encountered during the diagnosis of FB aspiration in patients with asthma exacerbation. In fact, FB aspiration leading to the occurrence of a PM in a child with asthma may go unnoticed and be wrongly attributed to asthma, which leads to delayed diagnosis as well as life-threatening and long-term complications.

## Case presentation

**Fig. 1:**
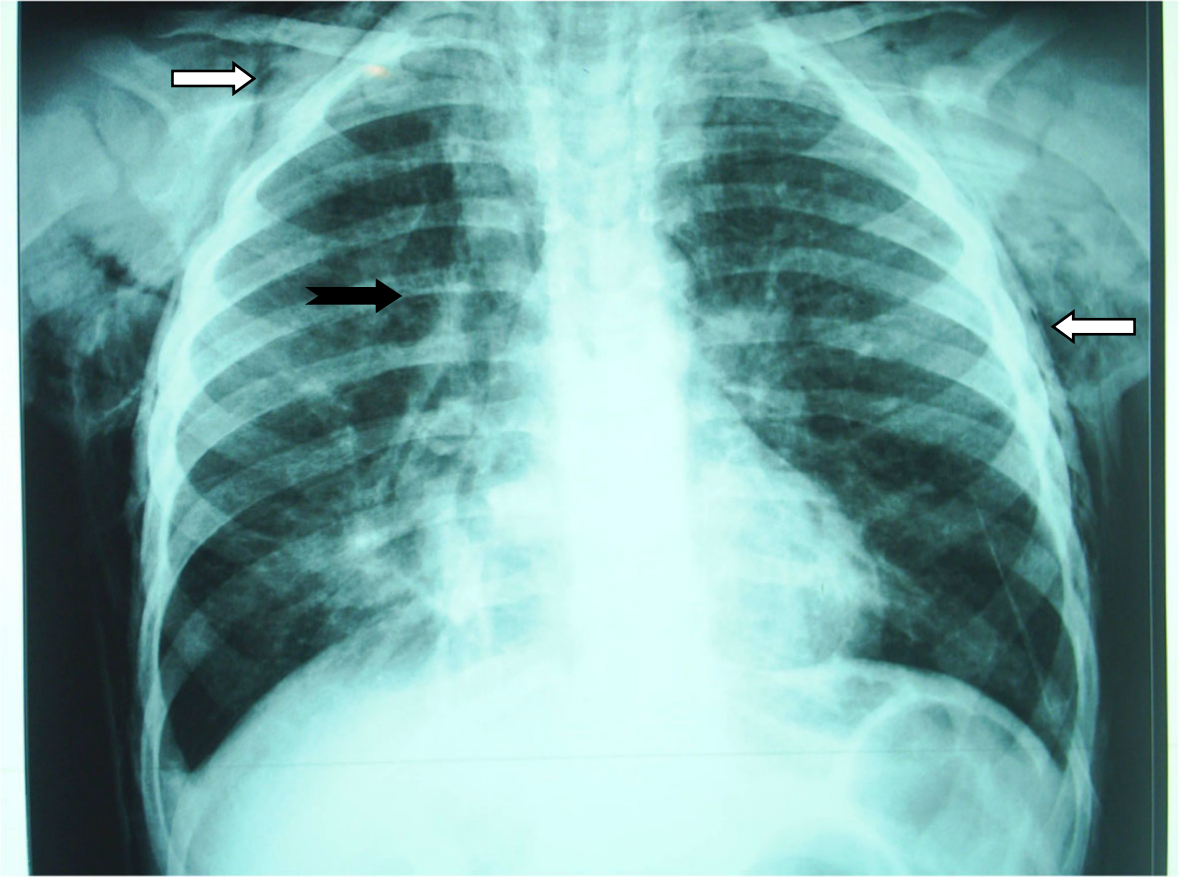
Radiograph of the chest showing subcutaneous emphysema (*white arrows*) and pneumomediastinum (*black arrow*), right basal pneumonia, and bilateral hyperinflation of the lung

**Fig. 2:**
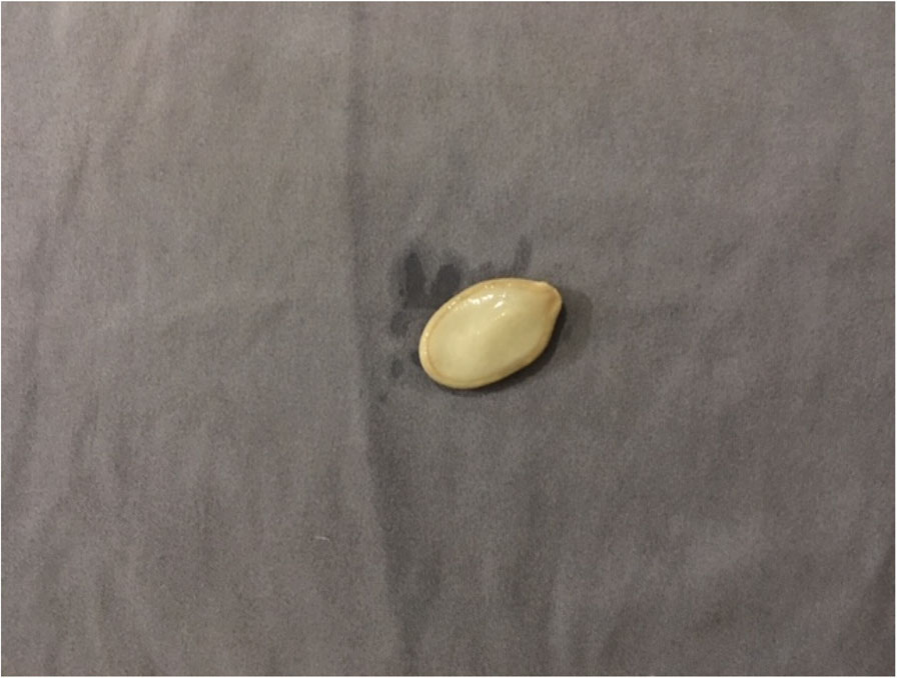
Foreign body (pumpkin seed) removed

A 6-year-old Moroccan boy with asthma was admitted to our emergency department because of acute dyspnea and persistent dry cough. Two days prior to his admission, he had fever, cough, and wheezing. He was initially treated with oral antibiotics (azithromycin 10 mg/kg/day) and nebulized salbutamol at his pediatrician’s office. However, owing to the worsening of his condition and the appearance of cervical swelling, he was referred to our department. Since age 3 years, the child had been diagnosed with intermittent asthma, had been well-monitored, and had been placed on outpatient treatment. Viral triggers were common with this patient. His family had an average socioeconomic level. Their home was airy and sunny without any pets. There was not any exposure to smoke from tobacco. The child had no particular pathological history. He had never been hospitalized for a severe crisis. He had nonfrequent recurrent wheezing episodes that occurred three to four times per year and were treated with bronchodilators and oral steroids when necessary. There was no family history of atopy. According to the parents, the patient had no history of food allergy, trauma, choking episode, FB aspiration, or any recent viral infection triggers for an acute asthma exacerbation. His physical examination showed the following: respiratory distress with perioral cyanosis, tachypnea (respiratory rate, 46/min) and hypoxia (oxygen saturation, 84% in room air), bilateral wheezing, and a cervical swelling with crepitations on the neck. His temperature was 38.3 °C. His hemodynamic state was stable (pulse rate, 110 beats/minute; blood pressure, 100/60 mmHg). The patient was conscious. The result of his neurological examination was normal. A chest x-ray showed SCE, PM, bilateral hyperinflation, and absence of radio-opaque FB (Fig. 1). The laboratory tests showed the following values: white blood cell count 17,000 cells/μl, hemoglobin 11.5 mg/dl, platelet count 220,000/μl, C-reactive protein 38 mg/L, urea 15 mg/dl, creatinine 0.8 mg/dl, and prothrombin time 100%. The boy was initially treated as having an acute asthma exacerbation complicated by PM and was administered oxygen, intravenous steroids (hydrocortisone 5 mg/kg/6 h), nebulized salbutamol, and amoxicillin-clavulanic acid (100 mg/kg/day). The evolution was made worse by respiratory distress (increase of respiratory rate at 54/min, decrease of oxygen saturation at 78% in room air) and extension of swelling that progressed from the neck to the face and shoulders. Owing to insufficient clinical and radiographic improvement under the appropriate treatment, the possibility of a differential diagnosis, especially that of FB aspiration, was considered. Thus, a rigid bronchoscopy (size 5.0) was performed under general anesthesia on the second day, which showed the FB (pumpkin seed) located at the entry of the right main bronchus (Fig. 2). After removal of the FB, a complete resolution of clinical signs was observed. The child was discharged to home the next day and was placed on outpatient treatment. Radiographic control after 10 days showed complete recovery. The evolution was normal after 2 years of follow-up with two asthma attacks treated on an outpatient basis with nebulized salbutamol.

## Discussion

This case report describes a 6-year-old boy who was admitted to our emergency department for acute dyspnea and persistent dry cough. On the basis of his past medical history of intermittent asthma, his physical examination, and results of his chest x-ray, the patient was initially treated as having an acute asthma exacerbation complicated by PM. Owing to insufficient clinical and radiographic improvement under the appropriate treatment, however, the possibility of a differential diagnosis, especially that of FB aspiration, was considered. A rigid bronchoscopy was performed, which showed an FB located at the right main bronchus. Our patient was younger (about 6 years old) than other patients in the literature, and no history of FB aspiration was reported.

SPM has been reported both as a consequence of an asthma exacerbation and as a sign of a first asthma attack in children. SPM may be associated with poorly controlled asthma. A majority of cases appear to occur in teenagers, and no obvious differences in incidence have been reported between the sexes [4]. Our patient was well controlled for intermittent asthma without any notable events. History taking, physical examination, and standard chest x-ray are most often diagnostic, and there is rarely a need for other investigation. Chest x-ray provides the right diagnosis in almost all cases [4, 5].

PM and SCE due to FB aspiration is a rare occurrence in the pediatric population. Only a few cases are described in the literature. One study in Taiwan showed that 81% of spontaneous PM was preceded by cough, and 50% of the patients with PM had asthma [1]. The main trigger factors in another study, analyzing the etiologies of spontaneous PM in children, were infections (43.2%) and asthma (21%), and only one child had FB aspiration [6]. In a review, Gasser *et al.* reported that the most frequent comorbidity in children was asthma (22.2%), and the most common trigger factors were bronchospasm (49%), cough (45.6%), vomiting (10.3%), and FB aspiration (8.3%) [5]. In a retrospective review of 155 children with FB aspiration, 9 patients (5.8%) had PM [7]. At the Children’s Medical Center in China, over a period of 10 years, no cases of spontaneous PM were found to be associated with FB aspiration [8]. In another review, performed in the United States, among 126 children with FB aspiration, only one case with PM was reported [2].

Even if the most common causes of spontaneous PM and SCE are asthma and infections, the possibility of FB aspiration should be considered, especially in patients under 3 years old, owing to multiple physiological, environmental, and developmental factors [9]. In a retrospective review of 55 children, Saliba *et al.* found that children with asthma were significantly more likely than children without asthma to have a negative bronchoscopy result. Their study concluded that a conservative approach cannot be justified in children with suspected asthma with possible FB aspiration [10]. Recently, a similar case report about three children with asthma was reported. The three children presenting with PM and SCE were treated initially as having acute asthma exacerbations, but they were found to have FB aspiration. These children were aged 16, 20, and 21 months. Through these cases, the authors highlight that physicians must remain vigilant and look for comorbidities in cases of asthma exacerbation, especially for FB aspiration, which should be excluded. Thus, a very high index of suspicion should be taken into account in cases with insufficient response to conventional asthma treatment and localized clinical signs [11]. Mohd Ariff *et al.* reported a case of an undiagnosed chicken meat aspiration in a 12-year-old boy with traumatic brain injury and a history of asthma. Their article emphasized that FB aspiration needs to be considered as one of the causes of postintubation difficult-to-ventilate scenarios. Prompt diagnosis and bronchoscope-assisted removal of FB is essential in reducing morbidity and mortality [12]. Note that the combination of acute asthma exacerbation and FB aspiration creates a pathophysiological condition that predisposes the appearance of PM, SCE, or even a pneumothorax (valve phenomenon). Because our patient was diagnosed with asthma and followed as such and had no recent history of FB aspiration as reported by the parents, he was initially treated as having an acute asthma exacerbation complicated by PM. There was no clinical or radiological evidence of FB aspiration. Owing to insufficient clinical improvement with the appropriate treatment, however, the possibility of a differential diagnosis, especially that of FB aspiration, was considered. So, a rigid bronchoscopy (size 5.0) was safely performed, and the FB was successfully removed.

Spontaneous PM generally resolves spontaneously within a few days. It requires a conservative treatment consisting of simple clinical monitoring, rest, and analgesics. Underlying disorders should also always be identified and treated [4, 13]. Cases of idiopathic SPM require diagnostic pulmonary function tests after the acute episode to establish whether the child has asthma [13]. The FB aspiration results in significant morbidity and mortality in children. Acute and chronic complications seem to occur in almost 15% of patients [14]. Early diagnosis and treatment are of utmost importance to prevent life-threatening and long-term complications. Close communication between the medical team (anesthesiologist, bronchoscopist, pediatrician, and assistants) is essential. Bronchoscopy is generally considered the gold standard of diagnosis. Rigid bronchoscopy under general anesthesia must be performed urgently, and the patient should be observed carefully. Supportive care should be administered with intravenous steroids and reflux medications [9]. But physicians must be aware of the risk of worsening bronchospasm in children with asthma who undergo bronchoscopy [11]. It has been shown that bronchoscopy under general anesthesia can be performed safely in children with difficult asthma if it is performed by experienced personnel [15]. Virtual bronchoscopy has also been proposed to minimize the risks of rigid bronchoscopy by shortening the operative time and providing information about the localization and size of the FB [16].

## Conclusions

Even if asthma exacerbation represents the most common cause of PM and SCE, the possibility of FB aspiration should always and carefully be considered in children with asthma as an important differential diagnosis, even in the absence of a history of FB aspiration.

## Abbreviations

FB: Foreign body
PM: Pneumomediastinum
SCE: Subcutaneous emphysema
